# Masculine depression and its problem behaviors: use alcohol and drugs, work hard, and avoid psychiatry!

**DOI:** 10.1007/s00406-023-01567-0

**Published:** 2023-02-28

**Authors:** Claudia von Zimmermann, Magdalena Hübner, Christiane Mühle, Christian P. Müller, Christian Weinland, Johannes Kornhuber, Bernd Lenz

**Affiliations:** 1 Department of Psychiatry and Psychotherapy, Friedrich-Alexander University Erlangen-Nürnberg (FAU), Schwabachanlage 6, 91054 Erlangen, Germany; 2 Department of Addictive Behavior and Addiction Medicine, Central Institute of Mental Health (CIMH), Medical Faculty Mannheim, Heidelberg University, Mannheim, Germany

**Keywords:** Male depression, Masculine depression, Alcoholism, Substance use, Drug use, Nicotine, Help seeking

## Abstract

The gender role influences vulnerability to mental illness. Substance use, even critical in scale, is perceived as masculine, just like hard (over-)work, while not seeking help. With the ongoing separation between gender and sex, masculine norms become more relevant also to females’ mental health. The male depression concept highlights the role of male symptoms in affective disorders. However, the empirical evidence is still limited. Here, we use the denomination ‘masculine depression’ to open the category for female patients and tested substance use patterns, health services’ utilization, and working hours as predictors in a case–control study of 163 depressed in-patients (44% women; masculine vs. non-masculine depression according to a median split of the Male Depression Rating Scale-22) and 176 controls (51% women). We assessed higher depression severity in patients with masculine (vs. non-masculine) depression. Masculine depression (vs. non-masculine depression and vs. no depression) was predicted by more frequent and critical use of alcohol (including binge drinking), tobacco, and illicit drugs, and by longer working times. Moreover, fewer health services contacts due to mental complaints during the previous year were associated with masculine (vs. non-masculine) depression. Alarmingly, even critical substance misuse was not significantly associated with more frequent health services contacts; however, the higher the depression severity, the more contacts the patients reported. Here, we provide evidence that patients with masculine depression are highly burdened and undertreated, which applies equally to female and male patients. This study identified promising targets to establish specialized care offers.

**Supplementary Information:**

The online version contains supplementary material available at 10.1007/s00406-023-01567-0.

## Introduction

Sex and gender influence the risk to develop illness. This effect is well known, e.g. for cardiovascular diseases; while sex has been shown to be more relevant for the etiopathogenesis, gender is more likely to influence disease manifestations and access to and quality of care [1, 2]. Such mechanisms also contribute to mental disorders like depression [3, 4]. For a long time, it has been assumed that females are twice as likely to develop depression than males [5]. However, applying newer and more specific screening tools in the context of research efforts on the Male Depression revealed a similarly high prevalence of depression in both sexes [6, 7]. Those screening tools are for example the Gotland Male Depression Scale (GMDS) [8], the Male Depression Risk Scale 22 (MDRS-22) [9], and the Gender-Sensitive Depression Screening (GSDS) [10, 11]. They survey so-called masculine (i.e., more externalizing symptoms) like anger, aggression, distraction, avoidance, emotional suppression, irritability, substance use, and risk-seeking behavior, which are typical for male depression [9, 12]. Interestingly, females can also be affected by the male depression subtype [13]. A greater exposure to chronic stressors [13] due to the burdens associated with pursuing a career and raising a family [14, 15] have been postulated to be reasons for masculine depression in women. With the ongoing separation between sex and gender, gender roles – independently from sex – gain greater relevance for the individual’s illness risk. Masculine norms become more and more relevant for females and could thus also be responsible for so-called male depression symptoms in females. To deliberately include females, we choose to use the term “masculine depression” instead of “male depression” in the following.

Thus far, little is known about the risk factors for masculine depression. E.g. maladaptive early childhood schemas have been suggested as predictors [16]. New findings in this area have the potential to improve care for those affected via tailored help approaches. Here we investigated whether adherence to masculine norms is an indicator for masculine depression.

“Typically masculine norms” are for example winning, emotional control, risk-taking, violence, dominance, self-reliance, and primacy of work [17]. Those masculine norms also refer to substance use [18], even critical in scale, just like hard (over-)work [19], but not seeking help [20].

Substance abuse frequently co-occurs with Major Depressive Disorder (MDD) [21–23]. Patients with substance use disorder (SUD) often develop depressive symptoms and use alcohol to reduce psychological strain [24, 25], and vice versa, patients suffering from depression often report harmful and addictive substance use. Saha et al*.* [26] found a six-fold elevated risk for broadly defined mood disorder and drug dependence in depressed patients. Eberhard et al*.* [27] describe a comorbidity of 41% of risky alcohol use and affective disorder in an emergency psychiatric inpatient population of adolescents. The link between MDD and SUD suggests a common pathophysiology. Overlapping genetic liability of alcohol use disorder (AUD) and cannabis use with depressive disorder has been reported [21, 28–30]. Common dysregulation of the neutral sphingomyelinase has also been shown to mediate parts of the SUD-depression comorbidity [31]. Alcohol exerts different effects on mice with genetically induced depression than normal control animals. It normalizes sphingolipid- and monoamine deficits in the brain [32]. This may provide a biological base for potentially beneficial effects of alcohol in the self-management of mood disorders [33]. However, the reasons for the large overlap of depression and SUD are still insufficiently understood, and the literature remains controversial. Kelly et al*.* [34] recently published a large national survey of persons with depressive symptoms and showed that men and emerging adults are at disproportionately higher risks of AUD and binge drinking than either women or older adults. Alarmingly, it has been shown that the prevalence of alcohol drinking among women increased in the USA between 2001/2002 and 2012/2013 [35]. We recently found an overlap of masculine depression with Cluster B personality [36]. Cluster B personality disorders (narcissistic, borderline, dissocial, and histrionic personality disorders) are characterized by impulsive, dramatic, emotionally unstable, and erratic behaviors. Patients with such a personality disorder are also at an increased risk of AUD [37].

The so-called male norm “overwork” is commonly defined as 50 or more hours per week. This is much more often found in men than in women [19] and women with children more often leave those male-dominated occupations in comparison to men or childless women [38]. The harmful impact of overwork on mental health is well known [39–41]. Although Kuroda et al*.* [41] found increasing job satisfaction in people who work more than 55 h per week, they also showed an impairment of workers’ mental health [41]. Interestingly, Virtanen et al*.* [42] showed that working more than 55 h a week is associated with an excess risk of depression and anxiety in women, but not men. Work beyond the standard time norm is associated with an increased alcohol use in men and women equally [43].

Men’s claim to be able to work and to be efficient could also be linked to the norm “not seeking help”. Depressed men's help-seeking behavior has been described as negatively affected by masculine norms [20, 44] previously. Reasons therefore could be that weakness and need for help are believed not to be masculine and help-seeking implies loss of status, loss of control and autonomy, incompetence, dependence, and damage of identity [45]. Instead, men consume alcohol, become more often “workaholics”, and have a higher risk of suicide [45].

Magovcevic et al*.* [46] showed that men adhering to hegemonic masculine norms are more likely to exhibit externalizing symptoms than symptoms of prototypic depression, in comparison to males not adhering to those masculine norms following stressful life events. But masculine norms become more and more important, also for females’ causes of depression and symptoms. Accordingly, a cross-sectional study with 200 pregnant women showed an association between conformity to male gender norms and nonconformity to some female gender norms and an increased risk of suffering from depression [47]. Taken together, the masculine depression is an important, yet underinvestigated field. We still lack empirical evidence on the risk factors in both sexes. To the best of our knowledge, there is no in-depth study available on how masculine depression associates with use of alcohol, tobacco, and illicit drugs and how this comorbidity is related to working hours and utilization of health services.

### Aims of the study

Here, we analyzed whether masculine depression is associated with substance use per se, more frequent substance use, more critical substance use, longer working hours, and less use of health care, regardless of sex. We tested if use patterns of alcohol, tobacco, and illicit drugs and health services contacts due to physical and/or mental complaints differ between patients with masculine depression and patients with non-masculine depression (patients with high and those with low MDRS-22 scores) and whether these parameters predict MDRS-22 scores. We recruited a sex-balanced cohort of in-patients with moderate to severe depression according to the ICD-10 diagnostic criteria and applied a sex-specific median split to subclassify patients with masculine depression and patients with non-masculine depression. We also compared these groups of depressed patients with healthy control subjects.

## Methods

### Sample population

The data analyzed here were collected as part of the Masculine Depression Project [36, 48] aiming to gain more knowledge about the masculine depression. It was conducted as a prospective, open-label, comparative cohort study with one single data collection point per participant. From May 2017 to November 2019, we screened a total of 658 study subjects and included 170 patients and 176 healthy control subjects. Recruitment of participants was conducted by a medically trained team and took place between 7 and 10 am. Each visit lasted approximately 3–4 h and consisted of several parts. Participation requirements were: a minimum age of 18 years, a body mass index < 35 kg/m^2^, and written informed consent.

The patient population was recruited from the Department of Psychiatry and Psychotherapy at the Friedrich-Alexander-University Erlangen-Nürnberg (FAU) and the Clinic for Psychiatry, Addiction, Psychotherapy, and Psychosomatics at the Europakanal in Erlangen, Germany. Inclusion required an inpatient stay due to a moderate or severe depressive ICD-10 episode in one of the two clinics mentioned or depressive symptoms of a recurrent unipolar or bipolar affective disorder classified as moderate or severe according to ICD-10 [49]. Also the diagnostic criteria for depression according to DSM-5 [50] had to be fulfilled. Study recruitment had to take place during the first five days of hospitalization. Psychotic disorders led to exclusion from the study in the group of patients and in the healthy control group.

The healthy control subjects were recruited among individuals who had expressed interest in participating in studies at the University Hospital in Erlangen. They were recruited by distributing flyers and by online advertising via social platforms. Flyers were mainly distributed in Erlangen and Nuremberg. A telephone screening was conducted with those interested in the study. The inclusion and exclusion criteria were confirmed at this step. We used the exclusion criterion mentioned for the patients’ group and added the following criteria: regular intake of psychotropic drugs, a current psychiatric diagnosis according to ICD-10 (except for nicotine dependence), or a history of in-patient treatment. The control subjects received an expense allowance of 30 euros.

### Evaluation of the depression symptoms, substance use parameters, and health services contacts

The MDRS-22 [9] was utilized to capture the characteristics of the masculine depression. The MDRS-22 is a self-reported scale consisting of 22 items to assess predominantly externalizing symptoms which are assumed to be typical for masculine depression. Six domains including emotional suppression, drug use, alcohol use, anger and aggression, somatic symptoms, and risk-taking were assessed. The participants evaluated the items in comparison with the previous month. Every item is rated on a scale from 0 to 7. Higher values indicate a more frequent occurrence of symptoms. We calculated means; thus, values of 0 to 7 can be achieved for the MDRS-22 score [9, 51, 52]. Due to the lack of a validated German version at the time of recruitment, the version by Rice et al*.* [9] was translated into German by the study team (Supplementary Table 1). We found a Cronbach's alpha of 0.828 for the patient group (0.787 for the control group), showing a good internal consistency, similar to another recently published German MDRS-22 version by Walther et al*.* [53].

The Beck’s Depression Inventory-II (BDI-II) [54] was used to determine depression severity. We employed the Alcohol Use Disorder Identification Test (AUDIT) in the German version [55] (scores ≥ 8 and ≥ 20 indicate zone II and IV risk levels; [56]) and a questionnaire on binge drinking behavior to obtain information about alcohol consumption [57]. The study subjects were asked to indicate on how many occasions they had consumed ≥ 5, ≥ 10, and ≥ 15 standard drinks within two hours during the last two weeks and the last 24 months. A standard drink was defined as 330 mL of beer (5% alcohol), 140 mL of wine (12% alcohol), or 70 mL of a 25% (e.g., aperitif) or 40 mL of a 40% alcohol liquor (e.g., whiskey, gin, vodka). Smoking behavior was determined using the Fagerström Test for Nicotine Dependence (FTND) [58], which includes six items. The drug intake was recorded using a short questionnaire. The last four weeks and the entire lifetime were analyzed. The following substances were inquired: cannabis, opioids, cocaine, hallucinogens, PCP, and sedatives (including hypnotics, anxiolytics, and analgesics). Finally, the participants reported how often they had contacted a physician within the last six months because of physical complaints and within the last 12 months because of psychological complaints.

### Measurement of gamma-glutamyl transferase activity

The gamma-glutamyl transferase (GGT) activity was determined at the Central Laboratory of the Universitätsklinikum Erlangen, Germany (DIN EN ISO 15189 accredited).

### Statistical analyses

We used sex-separated median values of the MDRS-22 to divide the sample of depressed patients into a group of 81 “patients with masculine depression” (i.e., high MDRS-22 scores) and 82 “patients with non-masculine depression” (i.e., low MDRS-22 scores). Seven study subjects with missing data for the MDRS-22 were excluded.

SPSS for Windows 27.0 (SPSS Inc., Chicago, IL, USA) was used to analyze the data and GraphPad Prism 8.4.3 (Graph Pad Software Inc., San Diego, CA, USA) to visualize the results. Variation in frequencies was tested using χ^2^ tests (and we report P values from two-tailed Fisher’s exact test if at least one cell failed to reach an expected value of five observations). Correlations used the Pearson method. We employed Student’s *t*-tests for differences in two independent groups, and the statistics were adjusted when necessary, according to the Levene’s test. We used binary regression analyses with high vs. low MDRS-22 score groups as primary dependent variable and linear regression analyses with the MDRS-22 score as dependent variable and substance use parameters, number of health services contacts, GGT, or working times as predictors. We then analyzed how these parameters predicted the secondary dependent variables, high MDRS-22 score group vs. healthy control group and low MDRS-22 score group vs. healthy control group (binary regression analyses). The regression models included sex and age and the models with high vs. low MDRS-22 score groups or the MDRS-22 score as dependent variable the BDI-II scores. Furthermore, linear regression models were computed to identify predictors of health services contacts. We report B coefficients and validated the results using bias-corrected and accelerated bootstrap (1000 resamples). A P value < 0.05 was considered significant.

## Results

### Cohort characteristics

Relative to patients with non-masculine depression, patients with masculine depression were younger (mean age: 36.4 vs. 45.7 years) and less often married (28% vs. 48%) (Table 1). Patients with masculine depression showed also more months of employment during the previous year (8.1 vs. 6.2), more hours of employment per week (25.0 vs. 19.1), and higher BDI-II scores (37.3 vs. 28.7). See Table 1 for further comparisons between the patients’ groups and the healthy controls. Table 2 reports the descriptive characteristics of the groups in terms of substance use, health services contacts, and GGT activity. The MDRS-22 sum score correlated with BDI-II scores in the group of patients with non-masculine depression (*N* = 81, *r* = 0.495, *P* < 0.001), in the control subjects (*N* = 174, *r* = 0.603, *P* < 0.001), and in the total sample (*N* = 335, *r* = 0.783, *P* < 0.001), but not significantly in patients with masculine depression (*N* = 80, *r* = 0.161, *P* = 0.153). The MDRS-22 sum score did not significantly correlate with age: patients with masculine depression: *N* = 81, *r* =  – 0.149, *P* = 0.184; patients with non-masculine depression: *N* = 82, *r* = 0.049, *P* = 0.661; control subjects: *N* = 176, *r* = 0.060, *P* = 0.432; and total sample: *N* = 339, *r* =  – 0.023, *P* = 0.670. Moreover, the MDRS-22 sum score did not significantly differ between females and males (coded “2” and “1”): patients with masculine depression: *N* = 81, *t* = 0.757, *P* = 0.452; patients with non-masculine depression: *N* = 82, *t* =  – 0.554, *P* = 0.581; control subjects: *N* = 176, *t* = 1.158, *P* = 0.249; and total sample: *N* = 339, *t *= 1.384, *P* = 0.167.

**Table 1:** Cohort characteristics

	Patients with masculine depression (*N*_total_ = 81)			Patients with non-masculine depression (*N*_total_ = 82)			Healthy control subjects (*N*_total_ = 176)			Patients with masculine vs. patients with non-masculine depression		Patients with masculine depression vs. controls		Patients with non-masculine depression vs. controls	
	*N*	M/F	SD	*N*	M/F	SD	*N*	M/F	SD	χ^2^ or t	*P*	χ^2^ or t	*P*	χ^2^ or t	*P*
% Men	81	56		82	56		176	49		0.0	0.944	1.0	0.319	1.2	0.279
Age (years)	81	36.4	14.1	82	45.7	14.6	176	37.2	13.7	– 4.1	** < 0.001**	– 0.4	0.684	4.5	** < 0.001**
Months of employment during the previous year	77	8.1	4.9	76	6.2	5.6	173	8.2	4.9	2.3	**0.023**	– 0.1	0.894	– 2.7	**0.007**
Hours of employment per week	77	25.0	17.6	74	19.1	19.0	173	19.0	15.7	2.0	**0.049**	2.6	**0.012**	0.0	0.989
% Living in a current partnership	79	48		77	55		176	68		0.6	0.421	9.3	**0.002**	4.3	**0.038**
% Married	79	28		80	48		175	27		6.5	**0.011**	0.0	0.945	9.9	**0.002**
% Divorced	79	14		78	21		176	14		1.2	0.274	0.0	0.953	1.6	0.207
% At School	79	5		80	1		176	1		1.9	0.210	3.7	0.076	0.0	1.0
% In Vocational training	79	9		80	4		176	2		1.8	0.210	7.4	**0.011**	1.0	0.380
% At university	79	11		80	8		176	40		0.7	0.401	20.5	** < 0.001**	27.4	** < 0.001**
% In an employment relationship	79	51		80	39		176	52		2.3	0.132	0.0	0.874	3.7	0.054
% Self-Employed	79	6		80	6		176	11		0.0	1.0	1.6	0.211	1.6	0.201
% Unemployed	79	13		80	19		176	3		1.1	0.291	9.5	**0.004**	19.3	** < 0.001**
% Retired	79	10		80	20		176	3		3.0	0.082	4.7	**0.039**	19.3	** < 0.001**
BDI-II score	80	37.3	10.6	81	28.7	10.3	174	3.4	3.8	5.2	** < 0.001**	27.9	** < 0.001**	21.5	** < 0.001**
MDRS-22 score	81	2.6	0.8	82	1.1	0.4	176	0.4	0.4	15.2	** < 0.001**	24.2	** < 0.001**	14.7	** < 0.001**

The table shows the valid number of subjects analyzed (*N*), means (M) or relative frequencies (F), standard deviation (SD), and the results of χ^2^ / Fisher and Student's *t*-tests. *BDI-II* Beck’s Depression Inventory-II, *MDRS-22* Male Depression Rating Scale-22. *P* < 0.05 in bold

**Table 2:** Descriptive statistics of substance use parameters, health services contacts, and liver enzyme activity

	Patients with masculine depression (*N*_total_ = 81)			Patients with non-masculine depression (*N*_total_ = 82)			Healthy control subjects (*N*_total_ = 176)		
	*N*	M/F	SD	*N*	M/F	SD	*N*	M/F	SD
*Alcohol*									
AUDIT score	80	9.8	9.2	80	2.9	3.1	176	4.4	3.1
% AUDIT score (≥ 8)	80	44		80	5		176	15	
% AUDIT score (≥ 20)	80	20		80	1		176	0	
% Binge drinking Yes vs. No (2-week)	63	35		70	4		167	31	
Binge drinking frequency (2-week; drinks per week)	63	1.1	2.9	70	0.0	0.1	167	0.3	0.5
Binge drinking severity (2-week; drinks per week)	63	0.5	0.9	70	0.0	0.2	167	0.3	0.6
% Binge drinking Yes vs. No (24-month)	60	62		70	27		165	50	
Binge drinking frequency (24-month; drinks per week)	60	0.5	1.0	70	0.0	0.1	165	0.1	0.3
Binge drinking severity (24-month; drinks per week)	60	1.1	1.1	70	0.3	0.6	165	0.7	0.9
*Nicotine*									
% Cigarette smoking (yes)	81	47		81	30		175	6	
FTND score	81	2.0	2.7	81	1.0	2.0	175	0.1	0.4
*Drugs*									
% Use of sedative medication (4-week)	72	28		77	31		174	5	
% Use of cannabis (4-week)	74	26		77	5		174	3	
% Use of stimulants (4-week)	75	3		76	1		174	1	
% Use of opioids (4-week)	75	3		76	3		174	1	
% Use of cocaine (4-week; yes)	75	0		76	0		173	0	
% Use of hallucinogens/PCP (4-week)	75	3		76	0		173	0	
% Use of sedative medication (lifetime)	76	41		81	36		174	11	
% Use of cannabis (lifetime)	77	53		80	18		174	18	
% Use of stimulants (lifetime)	76	20		79	6		174	3	
% Use of opioids (lifetime)	78	12		79	3		176	1	
% Use of cocaine (lifetime)	78	9		79	1		175	2	
% Use of hallucinogens / PCP (lifetime)	78	17		79	3		174	2	
*Number of health services contacts*									
Due to physical complaints (6-month)	80	3.5	3.6	80	2.9	4.2	175	1.0	1.6
Due to mental complaints (12-month)	80	11.0	15.4	80	12.0	14.8	175	0.1	0.5
*GGT activity (U/L)*	81	33.1	30.1	82	28.3	21.8	176	25.8	38.7

The table shows the valid number of subjects analyzed (*N*), means (M) or relative frequencies (F) and standard deviation (SD). *AUDIT* Alcohol Use Disorder Identification Test, *FTND* Fagerström Test for Nicotine Dependence, *GGT* gamma-glutamyl transferase, *2-week* previous 2 weeks, *4-week* previous 4 weeks, *6-month* previous 6 months, *12-month* previous 12 months, *24-month* previous 24-months

### Substance use parameters

**Fig. 1:**
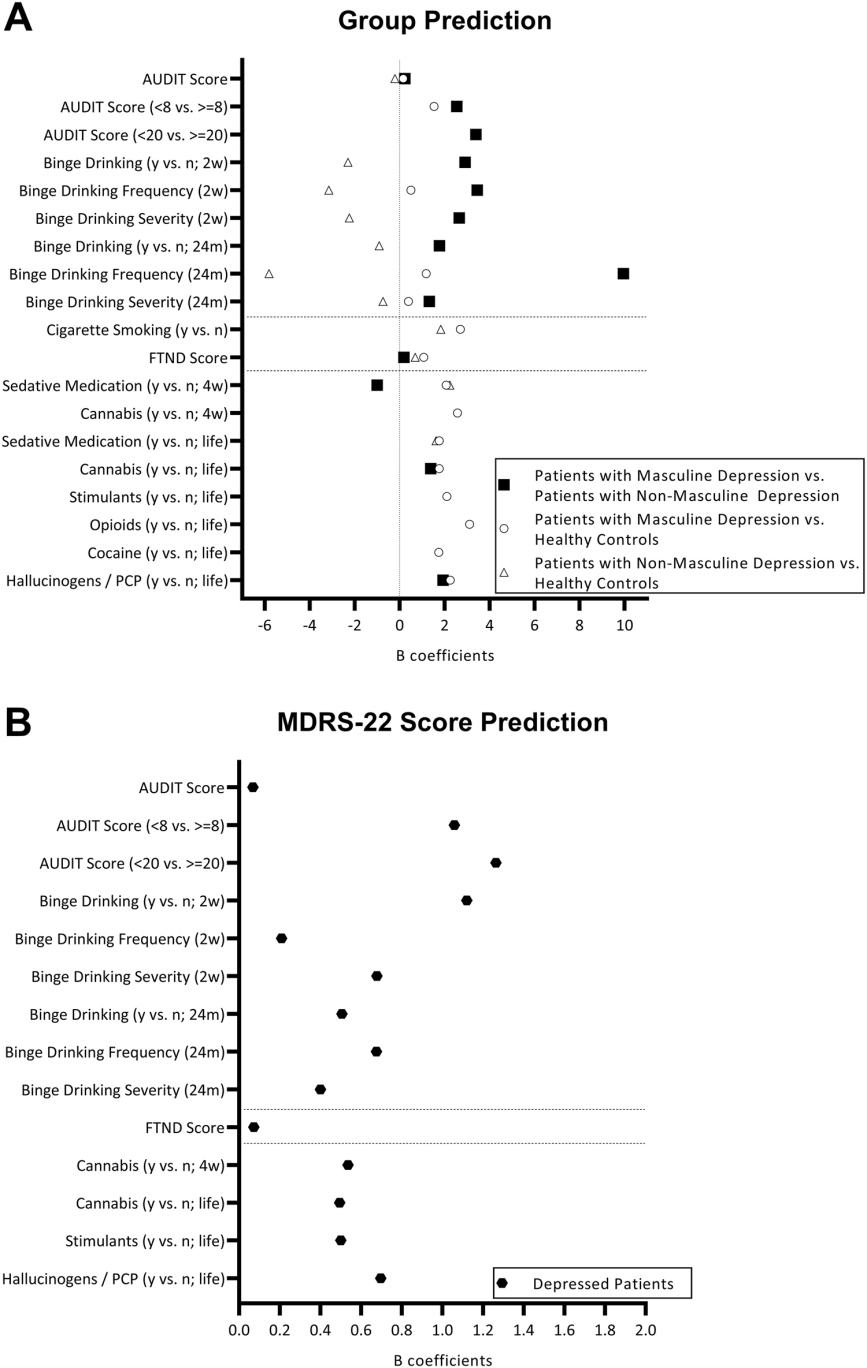
The figure shows the significant and bootstrap-validated B coefficients from binary logistic regression analyses to predict group (i.e., patients with masculine depression vs. patients with non-masculine depression vs. healthy controls; **A** Supplementary Tables S2, S4, S5) and linear regression analyses to predict MDRS-22 scores in the group of depressed patients (**B** Supplementary Table S3). MDRS-22, Male Depression Rating Scale 22; 2w, previous 2 weeks; 24 m, previous 24 months; 4w, previous 4 weeks; life, lifetime; y vs. n, yes vs. no

*Patients with masculine depression vs. patients with non-masculine depression:* The group of patients with masculine depression was predicted by higher AUDIT scores (B = 0.231, *P* < 0.001), an AUDIT score of at least 8 (vs. less than 8; B = 2.541, *P* < 0.001), an AUDIT score of at least 20 (vs. less than 20; B = 3.392, *P* = 0.003), binge drinking for both the 2-week and the 24-month periods (yes vs. no; B = 2.917, *P* < 0.001 and B = 1.771, *P* < 0.001), higher binge drinking frequency (2-week and 24-month periods: B = 3.454, *P* = 0.005 and B = 9.953, *P* = 0.001) and severity (2-week and 24-month periods: B = 2.658, *P* = 0.001 and B = 1.324, *P* < 0.001), higher FTND scores (B = 0.198, *P* = 0.012), and lifetime use of cannabis (B = 1.387, *P* = 0.002) and hallucinogens (B = 1.934, *P* = 0.024) (Fig. 1A, Supplementary Table S2). Moreover, the 4-week use of sedative medication was associated with the group of patients with non-masculine depression (B =  – 0.999, *P* = 0.037), and there was a statistical trend for an association between smoking and the group of patients with masculine depression (B = 0.679, *P* = 0.072). Overall, the group of patients with masculine depression was predicted by higher BDI-II scores (B from 0.088 to 0.127, *P* < 0.001) and younger age (B from  – 0.055 to  – 0.030, *P* from < 0.001 to 0.037; except for the model with 24-month binge drinking severity).

*MDRS-22 scores:* We found results similar to the above mentioned comparisons between patients with masculine depression and patients with non-masculine depression. In depressed patients, higher MDRS-22 scores were related to higher AUDIT scores (B = 0.068, *P* < 0.001), an AUDIT score of at least 8 (vs. less than 8; B = 1.059, *P* < 0.001), an AUDIT score of at least 20 (vs. less than 20; B = 1.264, *P* < 0.001), binge drinking behavior for both the 2-week and the 24-month periods (yes vs. no; B = 1.120, *P* < 0.001 and B = 0.506, *P* = 0.001), higher binge drinking frequency (2-week and 24-month periods: B = 0.209, *P* < 0.001 and B = 0.676, *P* < 0.001) and binge drinking severity (2-week and 24-month periods: B = 0.678, *P* < 0.001 and B = 0.400, *P* < 0.001), higher FTND scores (B = 0.073, *P* = 0.006), use of cannabis (4-week period, B = 0.536, *P* = 0.007; lifetime, B = 0.495, *P* = 0.002), use of stimulants (lifetime, B = 0.501, *P* = 0.012), and use of hallucinogens (lifetime, B = 0.697, *P *= 0.002) (Fig. 1B, Supplementary Table S3). Overall, higher BDI-II scores (B = from 0.034 to 0.044, *P* < 0.001) and younger age (B = from  – 0.018 to  – 0.008, *P* from < 0.001 to 0.046; except for 24-month binge drinking severity) predicted the MDRS-22 scores.

*Patients with masculine depression vs. healthy control subjects:* The group of patients with masculine depression (vs. the group of healthy controls) was related to higher AUDIT scores (B = 0.163, *P* < 0.001), an AUDIT score of at least 8 (vs. less than 8; B = 1.533, *P* < 0.001), higher 2-week and 24-month binge drinking frequency (B = 0.503, *P* = 0.006 and B = 1.188, *P* = 0.002) and 24-month severity (B = 0.392, *P* = 0.022), smoking behavior (B = 2.704, *P* < 0.001), higher FTND scores (B = 1.076, *P* < 0.001), 4-week use of sedative medication (B = 2.077, *P* < 0.001) and cannabis (B = 2.574, *P* < 0.001) and lifetime use of sedative medication (B = 1.760, *P* < 0.001), cannabis (B = 1.769, *P* < 0.001), stimulants (B = 2.105, *P* < 0.001), opioids (B = 3.114, *P *= 0.004), cocaine (B = 1.740, *P* = 0.014), and hallucinogens (B = 2.258, *P* < 0.001) (Fig. 1A, Supplementary Table S4). These models were not significantly affected by sex or age.

*Patients with non-masculine depression vs. healthy control subjects:* The group of patients with non-masculine depression (vs. the group of healthy controls) was linked to lower AUDIT scores (B =  – 0.201, *P* = 0.001), risk of binge drinking behavior (yes vs. no; 2-week and 24-month periods: B =  – 2.292, *P* < 0.001 and B =  – 0.909, *P* = 0.007), a lower frequency (2-week and 24-month periods: B =  – 3.144, *P* = 0.001 and B =  – 5.810, *P* = 0.007) and a milder severity (2-week and 24-month periods: B =  – 2.235, *P* < 0.001 and B =  – 0.732, *P* = 0.004) of binge drinking, smoking (B = 1.833, *P* < 0.001), a higher FTND score (B = 0.699, *P* = 0.001), and use of sedative medication (4-week period, B = 2.237, *P* < 0.001; lifetime, B = 1.622, *P* < 0.001) (Fig. 1A, Supplementary Table S5). Overall, patients with non-masculine depression were significantly predicted by higher age (B from 0.030 to 0.044, *P* from < 0.001 to 0.006).

### Number of health services contacts

*Patients with masculine depression vs. patients with non-masculine depression:* A lower number of health services contacts due to mental complaints predicted the group of patients with masculine depression (B =  – 0.025, *P* = 0.038), and there was a statistical trend for a higher GGT activity in patients with masculine depression (B = 0.015, *P* = 0.066). The models were again affected by BDI-II scores (B from 0.092 to 0.105, *P* < 0.001) and age (B from  – 0.055 to  – 0.051, *P* < 0.001) (Table 3).

**Table 3:** Regression analyses to predict groups of patients with masculine depression, patients with non-masculine depression, and healthy controls and Male Depression Rating Scale 22 scores

	Health services contacts, GGT activity, and working hours				Sex			BDI-II			Age		
	N	B	Wald / T	*P*	B	Wald / T	*P*	B	Wald / T	*P*	B	Wald / T	P
*Patients with masculine depression vs. patients with non-masculine depression*													
Health services contacts (Physical complaints)	159	0.038	0.6	0.424	0.570	2.1	0.145	0.092	21.3	< 0.001*	– 0.052	15.7	< 0.001*
Health services contacts (Mental complaints)	159	– 0.025	4.3	0.038*	0.694	3.0	0.083	0.105	23.9	< 0.001*	– 0.051	15.0	< 0.001*
GGT activity	161	0.015	3.4	0.066	0.466	1.3	0.246	0.094	21.8	< 0.001*	– 0.055	17.0	< 0.001*
Months of employment during the previous year	152	0.080	4.7	0.030*	0.682	2.9	0.090	0.101	22.5	< 0.001*	– 0.040	9.1	0.003*
Hours of employment per week	150	0.027	5.9	0.015*	0.514	1.6	0.210	0.114	24.7	< 0.001*	– 0.042	9.6	0.002*
*MDRS-22 scores*													
Health services contacts (Physical complaints)	159	0.025	1.5	0.141	0.296	2.2	0.032*	0.039	6.5	< 0.001*	– 0.018	– 4.2	< 0.001*
Health services contacts (Mental complaints)	159	– 0.012	– 2.8	0.005*	0.328	2.4	0.016*	0.045	7.4	< 0.001*	– 0.018	– 4.0	< 0.001*
GGT Activity	161	0.005	1.9	0.065	0.251	1.8	0.075	0.040	6.7	< 0.001*	– 0.019	– 4.2	< 0.001*
Months of employment during the previous year	152	0.024	1.9	0.063	0.326	2.3	0.021*	0.041	6.6	< 0.001*	– 0.014	– 2.9	0.004*
Hours of employment per week	150	0.007	2.0	0.053	0.265	1.9	0.059	0.044	7.0	< 0.001*	– 0.014	– 3.0	0.003*
*Patients with masculine depression vs. controls*													
Health services contacts (Physical complaints)	255	0.459	30.9	< 0.001*	0.417	1.9	0.173				– 0.007	0.4	0.548
Health services contacts (Mental complaints)	255	1.599	26.3	< 0.001*	0.299	0.4	0.525				– 0.017	0.9	0.338
GGT activity	257	0.005	1.6	0.206	0.194	0.5	0.483				– 0.007	0.5	0.499
Months of employment during the previous year	250	– 0.004	0.0	0.874	0.296	1.2	0.284				– 0.002	0.0	0.853
Hours of employment per week	250	0.022	6.1	0.014*	0.169	0.4	0.554				– 0.004	0.2	0.669
*Patients with non-masculine depression vs. controls*													
Health services contacts (Physical complaints)	255	0.259	14.1	< 0.001*	0.080	0.1	0.788				0.035	11.0	0.001*
Health services contacts (Mental complaints)	255	2.089	29.5	< 0.001*	0.143	0.1	0.802				0.043	5.1	0.024*
GGT activity	258	– 0.001	0.1	0.774	0.183	0.4	0.521				0.042	17.4	< 0.001*
Months of employment during the previous year	249	– 0.074	7.5	0.006*	0.213	0.5	0.465				0.035	12.2	< 0.001*
Hours of employment per week	247	– 0.006	0.4	0.515	0.280	0.9	0.343				0.037	13.1	< 0.001*

The table shows the valid number of subjects analyzed (*N*) and the results of binary logistic regression analyses (Patients with masculine depression vs. patients with non-masculine depression, patients with masculine depression vs. controls, patients with non-masculine depression vs. controls) and linear regression analyses (MDRS-22 score). *P* < 0.05 in bold, *also significant in bootstrap analysis. Coding: Patients with non-masculine depression = 0 vs. patients with masculine depression = 1, controls = 0 vs. patients with masculine / non-masculine depression = 1; females = 0 vs. males = 1. *GGT* gamma-glutamyl transferase, *MDRS-22* male depression rating scale 22

*MDRS-22 scores:* Similar to the above group prediction analysis, a higher MDRS-22 score was predicted by fewer previous health services contacts due to mental complaints (B =  – 0.012, *P* = 0.005) and there was a trend for a higher GGT activity (B = 0.005, *P* = 0.065) (Table 3). The models were influenced by BDI-II scores (B from 0.039 to 0.045, *P* < 0.001), age (B from  – 0.019 to  – 0.018, *P* < 0.001), and sex (B from 0.296 to 0.328, *P* from 0.016 to 0.032; except for GGT activity).

*Patients with masculine depression and patients with non-masculine depression vs. healthy control subjects:* A higher number of health service contacts for both physical and mental complaints was associated with the groups of patients with masculine depression and patients with non-masculine depression vs. healthy control subjects (physical issues: B = 0.459, *P* < 0.001, B = 0.259, *P* < 0.001; mental issues: 1.599, *P* < 0.001, B = 2.089, *P* < 0.001). Higher age predicted the patient group with non-masculine depression (B from 0.035 to 0.043, *P* from < 0.001 to 0.024) (Table 3) similar to the other models.

*Predictors of health care services contacts due to mental complaints:* We found that higher BDI-II scores indicative of more severe depression predict the group of patients with masculine vs. the group of patients with non-masculine depression and higher MDRS-22 scores in the group of depressed patients (Supplementary Tables S2 and S3). Simultaneously, fewer health services contacts due to mental complaints also predict the group of patients with masculine vs. the group of patients with non-masculine depression and lower MDRS-22 scores (Table 3). These results suggest that patients who are more severely affected by both depression symptoms and substance use request less frequently support for mental health complaints and may thus receive less intense treatment. To further explore underlying mechanisms, we analyzed how BDI-II score and substance use parameters were related to health services contacts in the group of depressed patients (Supplementary Table S6). As expected, higher BDI-II scores and use of sedative medication (4-week, lifetime) predicted more contacts (B = 0.350, *P* = 0.001; B = 6.543, *P* = 0.023; B = 6.792, *P* = 0.009). In contrast, alcohol, tobacco, and illicit drug use parameters were not significantly related to the number of health services contacts due to mental complaints in the prior year.

### Working hours

The sociodemographic characteristics showed significant differences in working hours (months of employment during the previous year, hours of employment per week) between the groups of patients with masculine depression, patients with non-masculine depression, and healthy control subjects (Table 1). Hence, we tested these parameters as predictors in regression analyses (Table 3) and found that more working hours for both parameters were associated with patients with masculine vs. patients with non-masculine depression (B = 0.080, *P* = 0.030 and B = 0.027, *P* = 0.015) and tended to predict the MDRS-22 score (B = 0.024, *P* = 0.063 and B = 0.007, *P* = 0.053). Relative to healthy controls, more hours of employment per week were associated with patients with masculine depression (B = 0.022, *P* = 0.014) and fewer months of employment during the previous year with patient with non-masculine depression (B =  – 0.074, *P* = 0.006). Overall, higher BDI-II scores and younger age predicted the group of patients with masculine depression vs. the group of patients with non-masculine depression and MDRS-22 scores (BDI-II scores: B from 0.041 to 0.114, *P *< 0.001; age: B from  – 0.042 to  – 0.014, P from 0.002 to 0.004); younger age also predicted healthy controls vs. patients with non-masculine depression (B from 0.035 to 0.037, *P* < 0.001). Notably, months of employment during the previous year and hours of employment per week did not significantly predict the number of health services contacts due to mental complaints in the prior year (Supplementary Table S6).

## Discussion

We investigated substance use patterns and health services contacts in masculine depression. We dichotomized a group of 163 depressed in-patients (44% women) into masculine vs. non-masculine depression (according to the Male Depression Rating Scale-22) and opened the category for female patients. We established that patients with masculine depression relative to those with non-masculine depression show more critical use patterns of alcohol, tobacco, cannabis, and hallucinogens. Simultaneously, patients with masculine depression report less frequent health services contacts due to mental complaints and less frequent use of sedatives. This finding is particularly important because patients with masculine depression are also burdened with increased depression severity. In conclusion, we show here that despite the more critical substance use patterns, patients with masculine depression are less likely to utilize health care services. This significant gap needs to be reduced in the future.

Here, patients with masculine depression showed more frequent and more problematic alcohol use than patients with non-masculine depression. This was applicable in terms of higher AUDIT scores, an AUDIT score of at least 8 (at least zone II risk level), an AUDIT score of at least 20 (zone IV risk level requesting referral to a specialist for diagnostic evaluation and treatment), as well as for binge drinking per se and higher binge drinking frequency and severity for both the 2-week and the 24-month periods. We also found a statistical trend for higher GGT activities in patients with masculine depression, which supports the self-rated higher AUDIT scores and higher binge drinking frequency and severity in this group of patients. Our validation analyses confirmed that higher MDRS-22 scores in the depressed patients were linked to more severe substance use, less health service utilization, and higher depression severity. Versus healthy control subjects, patients with masculine depression showed higher AUDIT scores and higher binge drinking frequency and severity. Interestingly, we found the opposite results for patients with non-masculine depression, i.e., a lower risk and less frequent and less severe binge drinking behavior compared to healthy controls.

Further support for this study’s findings comes from a previous investigation of the same study cohort showing more pronounced impulsive, borderline, and dissocial personality dimensions in patients with masculine depression than in patients with non-masculine depression [36]. Those personality traits are often associated with substance misuse [59]. The presence of SUD or antisocial behavior have been discussed as possible causes of externalizing behaviors [60, 61]. However, our study does not indicate whether SUD precede the masculine depression or vice versa. Future longitudinal studies are needed to elucidate which factors are causes and which ones represent consequences of the masculine depression.

Our results agree with the instrumentalization model of alcohol use. Alcohol can be instrumentalized in a way such that it is consumed in a controlled fashion. This in turn can lead to achievement of goals that would not be achievable or with a significantly higher work load without the drug. Humans and animals both use alcohol to self-manage depression and anxiety symptoms [62–64]. This may also apply to masculine depression. In particular, males reported alcohol use for self-management of masculine depression symptoms [65], which went along with a rejection of other medical treatment. Thereby, alcohol use is regarded as a “quick solution” [65].

We identified a significant gap in mental health care of patients with masculine depression. In this study, patients with masculine depression reported fewer health services contacts due to mental complaints and a lower rate of having used sedative medication during the previous 4-week period relative to those with non-masculine depression. These data suggest that patients with masculine depression have worse access to health service and/or show less help-seeking behavior. Here, higher BDI-II scores predicted more health services contacts due to mental complaints. However, the substance use parameters (except for sedative medication) failed to do so consistently.

Several reasons might account for this health care gap. A huge German survey showed that women more often consult psychiatrists or psychotherapists than men [66]. These differences may be driven by the masculine role model, which inhibits help-seeking behavior [67]. Such a mechanism might also explain this study’s findings that patients with masculine depression (both women and men) show fewer health services contacts. Further support for this assumption comes from our observation that patients with masculine depression reported more months of employment during the previous year as well as more hours of employment per week than patients with non-masculine depression. Versus healthy control subjects, patients with masculine depression even showed a higher number of hours of employment per week, while employment rates were not significantly different to the healthy controls. This finding is again in line with the masculine role model with, e.g., greater willingness to work (even to exhaustion) and higher avoidance of demonstrating weakness, e.g., in the form of a sick leave. Apparently, this also applies to women in the group of patients with masculine depression. Although this was outside the study objectives, our data indicate that the masculine depression is related to a male role model which does not allow oneself to show any weakness.

The results might also indicate that longer working hours promote externalizing symptoms of depression. Möller-Leimkühler et al*.* [13] assumed that masculine depression is highly prevalent in female university students as a result of exposure to chronic stressors. The combination of the higher risk for cluster B personality traits [36] and the higher consumption of alcohol identified here could lead to worse therapy outcomes [59]. This study’s data might also indicate that depressed patients are not sufficiently screened for their substance use and/or diagnosed with SUD. Future research is needed to enlighten the definite mechanisms underlying the health care gap identified here for patients with masculine depression.

The MDRS-22 correlated with the BDI-II score in the control group and the non-masculine depression group, but not significantly in patients with masculine depression. This lack of a significant correlation in patients with masculine depression is in line with the assumption that patients with masculine depression report only unreliably about typical depression symptoms. In our cohort, patients with masculine depression were younger than patients with non-masculine depression. These results agree with Kelly et al*.* [34] who found higher rates of comorbid suicidal ideation, AUD, and binge drinking in adults younger than 35 years than in older ones.

### Strengths and limitations

Major strengths of this project include the sex-balanced and large study cohort and the inclusion of relevant influencing factors such as sex, BDI-II, and age in the statistical models. The study is limited by the associational study design, which does not allow for conclusions regarding causality or directionality. Future longitudinal research is needed. We analyzed only depressed in-patients diagnosed according to the ICD-10. Individuals with masculine depression and lower depression severity might have been missed because less help-seeking behavior is assumed in patients with masculine depression. Future studies should also investigate the effects of depression history and antidepressants on masculine depression. The here employed median split method is limited due to its dependency on the specific cohort. Certainly, future research is needed to determine how to best distinguish between patients with masculine vs. patients with non-masculine depression.

## Conclusion

This study established that the masculine depression is related to more frequent and more critical use of alcohol, tobacco, and illicit drugs and simultaneously to less frequent health services contacts due to mental complaints and lower rates of use of sedative medication. Thus, we identify a significant gap in utilization of health services. Our results highlight the need for specialized and low-threshold help offers at an early stage of affective disorders related to the masculine depression.

### Supplementary Information

Below is the link to the electronic supplementary material.

Supplementary file1 (DOCX 93 KB)

## Data Availability

Data are available upon request.
