# Supplementary material for: Masculine depression and its problem behaviors: use alcohol and drugs, work hard, and avoid psychiatry!
Source: Eur Arch Psychiatry Clin Neurosci. 2023 Feb 28;274(2):321–33. doi: 10.1007/s00406-023-01567-0 (PMC10914846; doi:10.1007/s00406-023-01567-0)
Supplement: Supplementary file 1 — Supplementary file1 (DOCX 93 KB) [file 406_2023_1567_MOESM1_ESM.docx]

**Supplementary Material**

**Masculine Depression and Its Problem Behaviors: Use Alcohol and Drugs, Work Hard, and Avoid Psychiatry!**

Claudia von Zimmermann*^,1^, Magdalena Hübner^1^, Christiane Mühle^1^, Christian P. Müller^1,^ Christian Weinland^1^, Johannes Kornhuber^1,#^, Bernd Lenz^1,2,#^

^1^Department of Psychiatry and Psychotherapy, Friedrich-Alexander University Erlangen-Nürnberg (FAU), Germany.

^2^Department of Addictive Behavior and Addiction Medicine, Central Institute of Mental Health (CIMH), Medical Faculty Mannheim, Heidelberg University, Germany.

*Corresponding author. E-Mail: Claudia.von.Zimmermann@uk-erlangen.de

# These authors contributed equally.

**Supplementary Table S1** Items of the MDRS-22 translated into German

**Supplementary Table S2** Binary logistic regression to differentiate between patients with masculine depression and patients with non-masculine depression

**Supplementary Table S3** Linear regression to predict Male Depression Rating Scale 22 scores

**Supplementary Table S4** Binary logistic regression to differentiate between patients with masculine depression and healthy controls

**Supplementary Table S5** Binary logistic regression to differentiate between patients with non-masculine depression and healthy controls

**Supplementary Table S6** Linear regression to predict number of health services contacts in depressed patients

| **Supplementary Table S1** Items of the MDRS-22 translated into German |
| --- |
| 1. Ich habe meine negativen Gefühle unterdrückt.  2. Ich habe versucht meine Niedergeschlagenheit zu verdrängen.  3. Ich habe meine Probleme verborgen.  4. Ich habe alles alleine lösen müssen.  5. Ich habe Drogen genommen, um zurecht zu kommen.  6. Drogengebrauch hat mir vorrübergehende Erleichterung verschafft.  7. Ich habe Drogen beschafft.  8. Ich habe Alkohol gebraucht, um herunterzukommen.  9. Ich habe leichten Zugang zu Alkohol gebraucht.  10. Ich trank mehr Alkohol als sonst.  11. Ich habe mich durchs Trinken nicht mehr so schlecht gefühlt.  12. Ich verhielt mich anderen gegenüber verbal aggressiv.  13. Ich bin andere verbal angegangen, obwohl ich nicht provoziert worden bin.  14. Es war schwierig meine Wut/Ärger unter Kontrolle zu haben.  15. Ich habe in Situationen aggressiv überreagiert.  16. Ich hatte unerklärliche Schmerzen.  17. Ich hatte Magenschmerzen.  18. Ich hatte regelmäßige Kopfschmerzen.  19. Ich litt häufiger unter Sodbrennen als sonst.  20. Ich bin gefährlicher oder aggressiver Auto gefahren.  21. Ich habe aufgehört über die Folgen meiner Handlungen nachzudenken.  22. Ich habe unnötige Risiken in Kauf genommen. |
| The table shows the translated German version of the Male Depression Rating Scale-22 (MDRS-22) [1]. |

| **Supplementary Table S2** Binary logistic regression to differentiate between patients with masculine depression and patients with non-masculine depression | | | | | | | | | | | | | |
| --- | --- | --- | --- | --- | --- | --- | --- | --- | --- | --- | --- | --- | --- |
|  |  | Substance Use Parameter | | | Sex | | | BDI-II | | | Age | | |
|  | N | B | Wald | P | B | Wald | P | B | Wald | P | B | Wald | P |
| **Dependent Variable: Patients with Masculine Depression vs. Patients with Non-Masculine Depression** |  |  |  |  |  |  |  |  |  |  |  |  |  |
| **Alcohol** |  |  |  |  |  |  |  |  |  |  |  |  |  |
| AUDIT Score | 159 | 0.231 | 17.0 | **< 0.001*** | 0.365 | 0.6 | 0.422 | 0.111 | 20.9 | **< 0.001*** | -0.036 | 5.6 | **0.018*** |
| AUDIT Score (< 8 vs. ≥ 8) | 159 | 2.541 | 17.5 | **< 0.001*** | 0.294 | 0.5 | 0.485 | 0.091 | 17.6 | **< 0.001*** | -0.038 | 7.2 | **0.007*** |
| AUDIT Score (< 20 vs. ≥ 20) | 159 | 3.392 | 9.0 | **0.003*** | 0.725 | 3.0 | 0.082 | 0.102 | 22.3 | **< 0.001*** | -0.047 | 11.7 | **0.001*** |
| Binge Drinking (Yes vs. No; 2-week) | 132 | 2.917 | 13.5 | **< 0.001*** | 0.265 | 0.3 | 0.566 | 0.109 | 18.5 | **< 0.001*** | -0.044 | 8.7 | **0.003*** |
| Binge Drinking Frequency (2-week) | 132 | 3.454 | 7.9 | **0.005*** | 0.326 | 0.5 | 0.483 | 0.107 | 17.7 | **< 0.001*** | -0.045 | 9.0 | **0.003*** |
| Binge Drinking Severity (2-week) | 132 | 2.658 | 10.8 | **0.001*** | 0.252 | 0.3 | 0.585 | 0.108 | 18.3 | **< 0.001*** | -0.044 | 8.3 | **0.004*** |
| Binge Drinking (Yes vs. No; 24-month) | 129 | 1.771 | 12.8 | **< 0.001*** | 0.259 | 0.3 | 0.576 | 0.111 | 18.5 | **< 0.001*** | -0.038 | 6.0 | **0.014*** |
| Binge Drinking Frequency (24-month) | 129 | 9.953 | 11.3 | **0.001*** | 0.304 | 0.4 | 0.529 | 0.127 | 18.8 | **< 0.001*** | -0.037 | 5.3 | **0.022*** |
| Binge Drinking Severity (24-month) | 129 | 1.324 | 13.6 | **< 0.001*** | 0.105 | < 0.1 | 0.824 | 0.110 | 18.4 | **< 0.001*** | -0.031 | 3.7 | 0.055 |
| **Nicotine** |  |  |  |  |  |  |  |  |  |  |  |  |  |
| Cigarette Smoking (Yes vs. No) | 161 | 0.679 | 3.2 | 0.072 | 0.684 | 3.0 | 0.083 | 0.092 | 20.8 | **< 0.001*** | -0.051 | 15.1 | **< 0.001*** |
| FTND Score | 161 | 0.198 | 6.3 | **0.012*** | 0.696 | 3.0 | 0.082 | 0.095 | 21.1 | **< 0.001*** | -0.053 | 15.7 | **< 0.001*** |
| **Drugs** (Yes vs. No) |  |  |  |  |  |  |  |  |  |  |  |  |  |
| Sedative Medication (4-week) | 148 | -0.999 | 4.4 | **0.037*** | 0.567 | 1.8 | 0.179 | 0.115 | 23.4 | **< 0.001*** | -0.055 | 15.0 | **< 0.001*** |
| Cannabis (4-week) | 150 | 1.179 | 3.5 | 0.062 | 0.599 | 2.1 | 0.148 | 0.097 | 20.1 | **< 0.001*** | -0.044 | 10.0 | **0.002*** |
| Stimulants (4-week) | 150 | -0.093 | 0.0 | 0.948 | 0.615 | 2.3 | 0.133 | 0.102 | 22.2 | **< 0.001*** | -0.054 | 15.4 | **< 0.001*** |
| Opioids (4-week) | 150 | -1.195 | 0.9 | 0.350 | 0.656 | 2.5 | 0.114 | 0.106 | 22.8 | **< 0.001*** | -0.053 | 15.3 | **< 0.001*** |
| Cocaine (4-week) | 150 | - | - | - | 0.616 | 2.3 | 0.132 | 0.102 | 22.2 | **< 0.001*** | -0.054 | 15.5 | **< 0.001*** |
| Hallucinogens / PCP (4-week) | 150 | 19.773 | 0.0 | 0.999 | 0.639 | 2.4 | 0.119 | 0.100 | 21.7 | **< 0.001*** | -0.052 | 14.7 | **< 0.001*** |
| Sedative Medication (lifetime) | 156 | -0.319 | 0.6 | 0.430 | 0.573 | 2.1 | 0.151 | 0.091 | 18.9 | **< 0.001*** | -0.052 | 15.5 | **< 0.001*** |
| Cannabis (lifetime) | 156 | 1.387 | 9.8 | **0.002*** | 0.648 | 2.5 | 0.116 | 0.091 | 19.2 | **< 0.001*** | -0.030 | 4.4 | **0.037*** |
| Stimulants (lifetime) | 154 | 0.794 | 1.8 | 0.181 | 0.634 | 2.5 | 0.112 | 0.088 | 19.0 | **< 0.001*** | -0.044 | 11.5 | **0.001*** |
| Opioids (lifetime) | 156 | 1.140 | 1.5 | 0.214 | 0.669 | 2.9 | 0.091 | 0.090 | 20.3 | **< 0.001*** | -0.047 | 12.9 | **< 0.001*** |
| Cocaine (lifetime) | 156 | 1.397 | 1.2 | 0.271 | 0.692 | 3.1 | 0.081 | 0.089 | 19.6 | **< 0.001*** | -0.047 | 13.3 | **< 0.001*** |
| Hallucinogens / PCP (lifetime) | 156 | 1.934 | 5.1 | **0.024*** | 0.648 | 2.6 | 0.107 | 0.100 | 21.8 | **< 0.001*** | -0.045 | 11.4 | **0.001*** |
| The table shows the valid number of subjects analyzed (N) and the results of binary logistic regression analyses. AUDIT, Alcohol Use Disorder Identification Test; FTND, Fagerström Test for Nicotine Dependence; 2-week, previous 2 weeks; 4-week, previous 4 weeks; 24-month previous 24-months. P < 0.05 in bold, *also significant in bootstrap analysis. Coding: Patients with Non-Masculine Depression = 0 vs. Patients with Masculine Depression = 1; Females = 0 vs. Males = 1; AUDIT score < 8 = 0 vs. ≥ 8 = 1, < 20 = 0 vs. ≥ 20 = 1; Drug Use No = 0 vs. Yes = 1. | | | | | | | | | | | | | |

| **Supplementary Table S3** Linear regression to predict Male Depression Rating Scale 22 scores | | | | | | | | | | | | | |
| --- | --- | --- | --- | --- | --- | --- | --- | --- | --- | --- | --- | --- | --- |
|  |  | Substance Use Parameter | | | Sex | | | BDI-II | | | Age | | |
|  | N | B | T | P | B | T | P | B | T | P | B | T | P |
| **Dependent Variable: Male Depression Rating Scale 22 scores** |  |  |  |  |  |  |  |  |  |  |  |  |  |
| **Alcohol** |  |  |  |  |  |  |  |  |  |  |  |  |  |
| AUDIT Score | 159 | 0.068 | 9.3 | **< 0.001*** | 0.177 | 1.6 | 0.115 | 0.039 | 7.9 | **< 0.001*** | -0.008 | -2.2 | **0.030*** |
| AUDIT Score (< 8 vs. ≥ 8) | 159 | 1.059 | 7.5 | **< 0.001*** | 0.123 | 1.0 | 0.312 | 0.034 | 6.4 | **< 0.001*** | -0.009 | -2.2 | **0.033*** |
| AUDIT Score (< 20 vs. ≥ 20) | 159 | 1.264 | 6.6 | **< 0.001*** | 0.300 | 2.5 | **0.015*** | 0.040 | 7.5 | **< 0.001*** | -0.013 | -3.3 | **0.001*** |
| Binge Drinking (Yes vs. No; 2-week) | 132 | 1.120 | 6.8 | **< 0.001*** | 0.172 | 1.3 | 0.191 | 0.040 | 7.0 | **< 0.001*** | -0.013 | -3.1 | **0.002*** |
| Binge Drinking Frequency (2-week) | 132 | 0.209 | 6.8 | **< 0.001*** | 0.173 | 1.3 | 0.189 | 0.035 | 6.1 | **< 0.001*** | -0.016 | -3.9 | **< 0.001*** |
| Binge Drinking Severity (2-week) | 132 | 0.678 | 6.8 | **< 0.001*** | 0.148 | 1.1 | 0.260 | 0.038 | 6.6 | **< 0.001*** | -0.013 | -3.1 | **0.003*** |
| Binge Drinking (Yes vs. No; 24-month) | 129 | 0.506 | 3.3 | **0.001*** | 0.248 | 1.7 | 0.099 | 0.044 | 6.7 | **< 0.001*** | -0.013 | -2.5 | **0.013*** |
| Binge Drinking Frequency (24-month) | 129 | 0.676 | 7.6 | **< 0.001*** | 0.255 | 2.0 | **0.047** | 0.042 | 7.5 | **< 0.001*** | -0.011 | -2.5 | **0.014*** |
| Binge Drinking Severity (24-month) | 129 | 0.400 | 4.8 | **< 0.001*** | 0.178 | 1.2 | 0.221 | 0.042 | 6.7 | **< 0.001*** | -0.009 | -1.8 | 0.083 |
| **Nicotine** |  |  |  |  |  |  |  |  |  |  |  |  |  |
| Cigarette Smoking (Yes vs. No) | 161 | 0.263 | 2.0 | 0.052 | 0.320 | 2.4 | **0.019** | 0.039 | 6.5 | **< 0.001*** | -0.017 | -3.9 | **< 0.001*** |
| FTND Score | 161 | 0.073 | 2.8 | **0.006*** | 0.309 | 2.3 | **0.022*** | 0.039 | 6.6 | **< 0.001*** | -0.017 | -4.0 | **< 0.001*** |
| **Drugs** (Yes vs. No) |  |  |  |  |  |  |  |  |  |  |  |  |  |
| Sedative Medication (4-week) | 148 | -0.067 | -0.4 | 0.673 | 0.314 | 2.1 | **0.033*** | 0.042 | 6.5 | **< 0.001*** | -0.017 | -3.8 | **< 0.001*** |
| Cannabis (4-week) | 150 | 0.536 | 2.7 | **0.007*** | 0.286 | 2.0 | **0.042*** | 0.039 | 6.3 | **< 0.001*** | -0.013 | -2.9 | **0.004*** |
| Stimulants (4-week) | 150 | -0.432 | -0.9 | 0.376 | 0.301 | 2.1 | **0.035*** | 0.042 | 6.7 | **< 0.001*** | -0.018 | -4.1 | **< 0.001*** |
| Opioids (4-week) | 150 | 1.049 | 2.5 | **0.014** | 0.267 | 1.9 | 0.058 | 0.039 | 6.1 | **< 0.001*** | -0.018 | -4.2 | **< 0.001*** |
| Cocaine (4-week) | 150 | - | - | - | 0.307 | 2.2 | **0.032*** | 0.042 | 6.7 | **< 0.001*** | -0.018 | -4.0 | **< 0.001*** |
| Hallucinogens / PCP (4-week) | 150 | 0.087 | 0.1 | 0.885 | 0.309 | 2.2 | **0.032*** | 0.042 | 6.7 | **< 0.001*** | -0.018 | -3.9 | **< 0.001*** |
| Sedative Medication (lifetime) | 156 | 0.083 | 0.6 | 0.561 | 0.277 | 2.0 | **0.048*** | 0.035 | 5.5 | **< 0.001*** | -0.017 | -4.0 | **< 0.001*** |
| Cannabis (lifetime) | 156 | 0.495 | 3.2 | **0.002*** | 0.297 | 2.2 | **0.032*** | 0.038 | 6.3 | **< 0.001*** | -0.010 | -2.0 | **0.046*** |
| Stimulants (lifetime) | 154 | 0.501 | 2.5 | **0.012*** | 0.237 | 1.7 | 0.084 | 0.034 | 5.5 | **< 0.001*** | -0.015 | -3.5 | **0.001*** |
| Opioids (lifetime) | 156 | 0.346 | 1.3 | 0.198 | 0.302 | 2.1 | **0.033*** | 0.039 | 6.2 | **< 0.001*** | -0.016 | -3.6 | **0.001*** |
| Cocaine (lifetime) | 156 | 1.053 | 3.5 | **0.001** | 0.319 | 2.3 | **0.020*** | 0.035 | 5.6 | **< 0.001*** | -0.016 | -3.7 | **< 0.001*** |
| Hallucinogens / PCP (lifetime) | 156 | 0.697 | 3.1 | **0.002*** | 0.308 | 2.3 | **0.025*** | 0.039 | 6.5 | **< 0.001*** | -0.014 | -3.2 | **0.002*** |
| The table shows the valid number of subjects analyzed (N) and the results of linear regression analyses. AUDIT, Alcohol Use Disorder Identification Test; FTND, Fagerström Test for Nicotine Dependence; 2-week, previous 2 weeks; 4-week, previous 4 weeks; 24-month previous 24-months. P < 0.05 in bold, *also significant in bootstrap analysis. Coding: Females = 0 vs. Males = 1; AUDIT score < 8 = 0 vs. ≥ 8 = 1, < 20 = 0 vs. ≥ 20 =1; Drug Use No = 0 vs. Yes = 1. | | | | | | | | | | | | | |

| **Supplementary Table S4** Binary logistic regression to differentiate between patients with masculine depression and healthy controls | | | | | | | | | | |
| --- | --- | --- | --- | --- | --- | --- | --- | --- | --- | --- |
|  |  | Substance Use Parameter | | | Sex | | | Age | | |
|  | N | B | Wald | P | B | Wald | P | B | Wald | P |
| **Dependent Variable: Patients with Masculine Depression vs. Healthy Controls** |  |  |  |  |  |  |  |  |  |  |
| **Alcohol** |  |  |  |  |  |  |  |  |  |  |
| AUDIT Score | 256 | 0.163 | 26.7 | **< 0.001*** | -0.146 | 0.2 | 0.634 | 0.005 | 0.3 | 0.613 |
| AUDIT Score (< 8 vs. ≥ 8) | 256 | 1.533 | 22.4 | **< 0.001*** | -0.071 | 0.1 | 0.810 | 0.003 | 0.1 | 0.772 |
| AUDIT Score (< 20 vs. ≥ 20) | 256 | 22.205 | 0.0 | 0.998 | 0.234 | 0.6 | 0.424 | -0.001 | 0.0 | 0.905 |
| Binge Drinking (Yes vs. No; 2-week) | 230 | 0.138 | 0.2 | 0.672 | 0.147 | 0.2 | 0.630 | -0.007 | 0.4 | 0.537 |
| Binge Drinking Frequency (2-week) | 230 | 0.503 | 7.4 | **0.006*** | -0.010 | 0.0 | 0.974 | -0.005 | 0.2 | 0.642 |
| Binge Drinking Severity (2-week) | 230 | 0.371 | 2.8 | 0.096 | 0.050 | 0.0 | 0.872 | -0.005 | 0.2 | 0.672 |
| Binge Drinking (Yes vs. No; 24-month) | 225 | 0.433 | 1.7 | 0.189 | 0.122 | 0.1 | 0.699 | -0.003 | 0.1 | 0.787 |
| Binge Drinking Frequency (24-month) | 225 | 1.188 | 9.9 | **0.002*** | 0.062 | 0.0 | 0.847 | 0.002 | 0.0 | 0.866 |
| Binge Drinking Severity (24-month) | 225 | 0.392 | 5.2 | **0.022*** | 0.046 | 0.0 | 0.886 | 0.001 | 0.0 | 0.926 |
| **Nicotine** |  |  |  |  |  |  |  |  |  |  |
| Cigarette Smoking (Yes vs. No) | 256 | 2.704 | 46.1 | **< 0.001*** | 0.269 | 0.8 | 0.386 | -0.009 | 0.6 | 0.421 |
| FTND Score | 256 | 1.076 | 20.8 | **< 0.001*** | 0.268 | 0.7 | 0.399 | -0.004 | 0.1 | 0.755 |
| **Drugs** (Yes vs. No) |  |  |  |  |  |  |  |  |  |  |
| Sedative Medication (4-week) | 246 | 2.077 | 21.8 | **< 0.001*** | 0.512 | 2.8 | 0.095 | -0.006 | 0.3 | 0.566 |
| Cannabis (4-week) | 248 | 2.574 | 22.3 | **< 0.001*** | 0.284 | 0.9 | 0.343 | 0.008 | 0.5 | 0.470 |
| Stimulants (4-week) | 249 | 1.496 | 1.5 | 0.226 | 0.230 | 0.7 | 0.409 | -0.006 | 0.4 | 0.548 |
| Opioids (4-week) | 249 | 1.559 | 1.6 | 0.208 | 0.231 | 0.7 | 0.408 | -0.007 | 0.5 | 0.487 |
| Cocaine (4-week) | 248 | - | - | - | 0.253 | 0.8 | 0.363 | -0.006 | 0.4 | 0.546 |
| Hallucinogens / PCP (4-week) | 248 | 22.167 | 0.0 | 0.999 | 0.310 | 1.2 | 0.270 | -0.004 | 0.2 | 0.695 |
| Sedative Medication (lifetime) | 250 | 1.760 | 26.6 | **< 0.001*** | 0.377 | 1.6 | 0.204 | -0.005 | 0.2 | 0.644 |
| Cannabis (lifetime) | 251 | 1.769 | 29.9 | **< 0.001*** | 0.177 | 0.4 | 0.549 | 0.014 | 1.5 | 0.221 |
| Stimulants (lifetime) | 250 | 2.105 | 15.1 | **< 0.001*** | 0.289 | 1.0 | 0.317 | 0.001 | 0.0 | 0.919 |
| Opioids (lifetime) | 254 | 3.114 | 8.5 | **0.004*** | 0.282 | 1.0 | 0.320 | 0.000 | 0.0 | 0.998 |
| Cocaine (lifetime) | 253 | 1.740 | 6.0 | **0.014*** | 0.329 | 1.4 | 0.238 | -0.001 | 0.0 | 0.911 |
| Hallucinogens / PCP (lifetime) | 252 | 2.258 | 14.0 | **< 0.001*** | 0.461 | 2.6 | 0.110 | 0.002 | 0.0 | 0.826 |
| The table shows the valid number of subjects analyzed (N) and the results of binary logistic regression analyses. AUDIT, Alcohol Use Disorder Identification Test; FTND, Fagerström Test for Nicotine Dependence; 2-week, previous 2 weeks; 4-week, previous 4 weeks; 24-month previous 24-months. P < 0.05 in bold, *also significant in bootstrap analysis. Coding: Healthy Controls = 0 vs. Patients with Masculine Depression = 1; Females = 0 vs. Males = 1; AUDIT score < 8 = 0 vs. ≥ 8 = 1, < 20 = 0 vs. ≥ 20 =1; Drug Use No = 0 vs. Yes = 1. | | | | | | | | | | |

| **Supplementary Table S5** Binary logistic regression to differentiate between patients with non-masculine depression and healthy controls | | | | | | | | | | |
| --- | --- | --- | --- | --- | --- | --- | --- | --- | --- | --- |
|  |  | Substance Use Parameter | | | Sex | | | Age | | |
|  | N | B | Wald | P | B | Wald | P | B | Wald | P |
| **Dependent Variable: Patients with Non-Masculine Depression vs. Healthy Controls** |  |  |  |  |  |  |  |  |  |  |
| **Alcohol** |  |  |  |  |  |  |  |  |  |  |
| AUDIT Score | 256 | -0.201 | 10.5 | **0.001*** | 0.442 | 2.2 | 0.137 | 0.034 | 10.8 | **0.001*** |
| AUDIT Score (< 8 vs. ≥ 8) | 256 | -1.062 | 3.4 | 0.067 | 0.292 | 1.0 | 0.312 | 0.036 | 13.0 | **< 0.001*** |
| AUDIT Score (< 20 vs. ≥ 20) | 256 | 22.353 | 0.0 | 1.000 | 0.142 | 0.3 | 0.617 | 0.041 | 16.5 | **< 0.001*** |
| Binge Drinking (Yes vs. No; 2-week) | 237 | -2.292 | 13.5 | **< 0.001*** | 0.360 | 1.4 | 0.245 | 0.033 | 9.0 | **0.003*** |
| Binge Drinking Frequency (2-week) | 237 | -3.144 | 10.2 | **0.001*** | 0.348 | 1.3 | 0.261 | 0.033 | 9.2 | **0.002*** |
| Binge Drinking Severity (2-week) | 237 | -2.235 | 13.0 | **< 0.001*** | 0.364 | 1.4 | 0.240 | 0.032 | 8.8 | **0.003*** |
| Binge Drinking (Yes vs. No; 24-month) | 235 | -0.909 | 7.3 | **0.007*** | 0.393 | 1.6 | 0209 | 0.031 | 8.4 | **0.004*** |
| Binge Drinking Frequency (24-month) | 235 | -5.810 | 7.3 | **0.007*** | 0.325 | 1.1 | 0.290 | 0.032 | 8.9 | **0.003*** |
| Binge Drinking Severity (24-month) | 235 | -0.732 | 8.1 | **0.004*** | 0.407 | 1.7 | 0.194 | 0.030 | 7.5 | **0.006*** |
| **Nicotine** |  |  |  |  |  |  |  |  |  |  |
| Cigarette Smoking (Yes vs. No) | 256 | 1.833 | 18.9 | **< 0.001*** | 0.310 | 1.1 | 0.299 | 0.036 | 12.1 | **< 0.001*** |
| FTND Score | 256 | 0.699 | 12.0 | **0.001*** | 0.318 | 1.1 | 0.291 | 0.037 | 12.5 | **< 0.001*** |
| **Drugs** (Yes vs. No) |  |  |  |  |  |  |  |  |  |  |
| Sedative Medication (4-week) | 251 | 2.237 | 25.4 | **< 0.001*** | 0.430 | 1.9 | 0.169 | 0.039 | 12.9 | **< 0.001*** |
| Cannabis (4-week) | 251 | 1.144 | 2.6 | 0.107 | 0.203 | 0.5 | 0.480 | 0.041 | 15.8 | **< 0.001*** |
| Stimulants (4-week) | 250 | 0.770 | 0.3 | 0.597 | 0.234 | 0.7 | 0.417 | 0.040 | 15.2 | **< 0.001*** |
| Opioids (4-week) | 250 | 1.025 | 0.7 | 0.410 | 0.250 | 0.7 | 0.388 | 0.039 | 14.3 | **< 0.001*** |
| Cocaine (4-week) | 249 | - | - | - | 0.248 | 0.7 | 0.390 | 0.040 | 15.5 | **< 0.001*** |
| Hallucinogens / PCP (4-week) | 249 | - | - | - | 0.248 | 0.7 | 0.390 | 0.040 | 15.5 | **< 0.001*** |
| Sedative Medication (lifetime) | 255 | 1.622 | 21.0 | **< 0.001*** | 0.193 | 0.4 | 0.514 | 0.044 | 17.3 | **< 0.001*** |
| Cannabis (lifetime) | 254 | 0.408 | 1.1 | 0.286 | 0.217 | 0.6 | 0.444 | 0.042 | 16.3 | **< 0.001*** |
| Stimulants (lifetime) | 253 | 0.771 | 1.3 | 0.247 | 0.209 | 0.5 | 0.463 | 0.038 | 14.3 | **< 0.001*** |
| Opioids (lifetime) | 255 | 1.087 | 0.8 | 0.386 | 0.238 | 0.7 | 0.406 | 0.038 | 14.3 | **< 0.001*** |
| Cocaine (lifetime) | 254 | -0.032 | 0.0 | 0.979 | 0.215 | 0.6 | 0.448 | 0.039 | 15.6 | **< 0.001*** |
| Hallucinogens / PCP (lifetime) | 253 | 0.224 | 0.1 | 0.804 | 0.271 | 0.9 | 0.340 | 0.039 | 15.2 | **< 0.001*** |
| The table shows the valid number of subjects analyzed (N) and the results of binary logistic regression analyses. AUDIT, Alcohol Use Disorder Identification Test; FTND, Fagerström Test for Nicotine Dependence; 2-week, previous 2 weeks; 4-week, previous 4 weeks; 24-month previous 24-months. P < 0.05 in bold, *also significant in bootstrap analysis. Coding: Healthy Controls = 0 vs. Patients with Non-Masculine Depression = 1; Females = 0 vs. Males = 1; AUDIT score < 8 = 0 vs. ≥ 8 = 1, < 20 = 0 vs. ≥ 20 =1; Drug Use No = 0 vs. Yes = 1. | | | | | | | | | | |

| **Supplementary Table S6** Linear regression to predict number of health services contacts in depressed patients | | | | | | | | | | |
| --- | --- | --- | --- | --- | --- | --- | --- | --- | --- | --- |
|  |  | BDI-II, Substance Use Parameter, Working Hours | | | Sex | | | Age | | |
|  | N | B | T | P | B | T | P | B | T | P |
| **Dependent Variable: Number of Health Services Contacts Due to Mental Complaints** |  |  |  |  |  |  |  |  |  |  |
| **BDI-II Score** | 159 | 0.350 | 3.2 | **0.001*** | 1.796 | 0.7 | 0.465 | -0.018 | -0.2 | 0.818 |
| **Alcohol** |  |  |  |  |  |  |  |  |  |  |
| AUDIT Score | 158 | -0.163 | -1.0 | 0.315 | -0.170 | -0.1 | 0.944 | -0.044 | -0.5 | 0.594 |
| AUDIT Score (< 8 vs. ≥ 8) | 158 | -1.968 | -0.7 | 0.502 | -0.195 | -0.1 | 0.936 | -0.038 | -0.5 | 0.648 |
| AUDIT Score (< 20 vs. ≥ 20) | 158 | -1.051 | -0.3 | 0.787 | -0.475 | -0.2 | 0.844 | -0.025 | -0.3 | 0.758 |
| Binge Drinking (Yes vs. No; 2-week) | 131 | -4.812 | -1.3 | 0.191 | 1.736 | 0.6 | 0.544 | -0.060 | -0.6 | 0.529 |
| Binge Drinking Frequency (2-week) | 131 | 0.205 | 0.3 | 0.763 | 1.077 | 0.4 | 0.707 | -0.038 | -0.4 | 0.687 |
| Binge Drinking Severity (2-week) | 131 | -1.321 | -0.6 | 0.553 | 1.464 | 0.5 | 0.611 | -0.049 | -0.5 | 0.605 |
| Binge Drinking (Yes vs. No; 24-month) | 128 | 0.208 | 0.1 | 0.946 | 0.513 | 0.2 | 0.861 | -0.066 | -0.7 | 0.508 |
| Binge Drinking Frequency (24-month) | 128 | -0.785 | -0.4 | 0.704 | 0.642 | 0.2 | 0.823 | -0.076 | -0.8 | 0.439 |
| Binge Drinking Severity (24-month) | 128 | 0.515 | 0.3 | 0.768 | 0.354 | 0.1 | 0.904 | -0.057 | -0.6 | 0.582 |
| **Nicotine** |  |  |  |  |  |  |  |  |  |  |
| Cigarette Smoking (Yes vs. No) | 160 | 1.369 | 0.6 | 0.582 | 0.024 | 0.0 | 0.992 | -0.029 | -0.4 | 0.723 |
| FTND Score | 160 | 0.082 | 0.2 | 0.868 | -0.030 | 0.0 | 0.990 | -0.031 | -0.4 | 0.703 |
| **Drugs** (Yes vs. No) |  |  |  |  |  |  |  |  |  |  |
| Sedative Medication (4-week) | 148 | 6.543 | 2.3 | **0.023*** | 1.510 | 0.6 | 0.568 | -0.029 | -0.3 | 0.732 |
| Cannabis (4-week) | 150 | 1.865 | 0.5 | 0.619 | 0.478 | 0.2 | 0.853 | -0.018 | -0.2 | 0.840 |
| Stimulants (4-week) | 150 | -6.756 | -0.7 | 0.463 | 0.245 | 0.1 | 0.924 | -0.042 | -0.5 | 0.621 |
| Opioids (4-week) | 150 | 7.837 | 1.0 | 0.323 | 0.208 | 0.1 | 0.936 | -0.038 | -0.5 | 0.650 |
| Cocaine (4-week) | 150 | - | - | - | 0.354 | 0.1 | 0.891 | -0.034 | -0.4 | 0.684 |
| Hallucinogens / PCP (4-week) | 150 | -12.333 | -1.1 | 0.275 | 0.007 | 0.0 | 0.998 | -0.047 | -0.6 | 0.579 |
| Sedative Medication (lifetime) | 155 | 6.792 | 2.6 | **0.009*** | 1.164 | 0.5 | 0.644 | -0.015 | -0.2 | 0.849 |
| Cannabis (lifetime) | 156 | -1.980 | -0.7 | 0.488 | -0.747 | -0.3 | 0.759 | -0.056 | -0.6 | 0.536 |
| Stimulants (lifetime) | 154 | -6.551 | -1.8 | 0.080 | -0.212 | -0.1 | 0.932 | -0.061 | -0.7 | 0.471 |
| Opioids (lifetime) | 156 | 0.638 | 0.1 | 0.896 | -0.210 | -0.1 | 0.933 | -0.032 | -0.4 | 0.702 |
| Cocaine (lifetime) | 156 | -3.022 | -0.5 | 0.612 | -0.284 | -0.1 | 0.909 | -0.036 | -0.4 | 0.665 |
| Hallucinogens / PCP (lifetime) | 156 | -5.502 | -1.3 | 0.196 | -0.428 | -0.2 | 0.864 | -0.058 | -0.7 | 0.486 |
| **Working Hours** |  |  |  |  |  |  |  |  |  |  |
| Months of Employment During the Previous Year | 152 | -0.059 | -0.2 | 0.808 | 0.212 | 0.1 | 0.934 | -0.035 | -0.4 | 0.692 |
| Hours of Employment per Week | 150 | -0.016 | -0.2 | 0.820 | 0.177 | 0.1 | 0.946 | -0.027 | -0.3 | 0.754 |
| The table shows the valid number of subjects analyzed (N) and the results of binary logistic regression analyses. AUDIT, Alcohol Use Disorder Identification Test; FTND, Fagerström Test for Nicotine Dependence; 2-week, previous 2 weeks; 4-week, previous 4 weeks; 24-month previous 24-months. P < 0.05 in bold, *also significant in bootstrap analysis. Coding: Females = 0 vs. Males = 1; AUDIT score < 8 = 0 vs. ≥ 8 = 1, < 20 = 0 vs. ≥ 20 =1; Drug Use No = 0 vs. Yes = 1. | | | | | | | | | | |

**References**

1. Rice SM, Fallon BJ, Aucote HM, Möller-Leimkühler AM (2013) Development and preliminary validation of the male depression risk scale: Furthering the assessment of depression in men. J Affect Disord 151:950-958. https://doi. org/10.1016/j.jad.2013.08.013
